# Dimensions Define Stability: Insertion Torque of Orthodontic Mini-Implants: A Comparative In Vitro Study

**DOI:** 10.3390/jcm14051752

**Published:** 2025-03-05

**Authors:** Cristian Liviu Romanec, Tinela Panaite, Irina Nicoleta Zetu

**Affiliations:** Department of Oral and Maxillofacial Surgery, Faculty of Dental Medicine, “Grigore. T. Popa” University of Medicine and Pharmacy, 16 Universitatii Str., 700115 Iasi, Romania; liviu.romanec@umfiasi.ro (C.L.R.); irina.zetu@umfiasi.ro (I.N.Z.)

**Keywords:** artificial bone (Sawbones^®^), diameter effect, insertion torque, in vitro implant analysis, implant stability, mini-implant, orthodontic anchorage, orthodontic implant

## Abstract

**Background**: Mini-implants have transformed orthodontic treatment by providing reliable anchorage and addressing challenges in anchorage control. This in vitro study aimed to compare the insertion torque (IT) values of three types of orthodontic mini-implants. The null hypothesis stated that no significant difference would be found in IT based on mini-implant type. **Methods**: We analyzed the mechanical ITs of 12 mini-implants categorized into four groups based on lengths (6, 8, 10, and 12 mm) and diameters (1.4, 1.6, and 2.0 mm). Mini-implants were inserted at a 90° angle into artificial bone (Sawbones^®^) without pre-drilling. The ANOVA and Tukey’s post hoc test assessed differences, and Spearman’s correlation evaluated relationships between IT, diameter, and length. **Results**: The Lomas Mondefit^®^ 2 × 8 mm mini-implant had the highest IT (35 N), while the Jeil 2 × 12 mm had the lowest. Torque correlated with diameter (ρ = 0.609, *p* = 0.047) and length (ρ = 0.890, *p* < 0.001). The ANOVA showed significant differences (*p* = 0.035), with Leone^®^ and Lomas Mondefit^®^ differing significantly (*p* = 0.029). Mini-implant dimensions strongly influence IT. **Conclusions**: Mini-implant diameter and length significantly influence IT, highlighting their importance in clinical applications for optimal stability and performance.

## 1. Introduction

Orthodontic mini-implants represent a significant advancement in dental anchorage, effectively improving the success rates of orthodontic treatments while minimizing long-term complications [1]. Their introduction has revolutionized anchorage control in orthodontics, offering a minimally invasive, cost-effective, and versatile solution [2]. The widespread use of mini-implants is largely attributed to these advantages; however, the improper load distribution or biomechanical factors can affect primary stability and potentially lead to complications.

The efficiency of primary stability, assessed using IT, is a critical factor in determining the durability of the mini-implant during orthodontic treatment. To reduce the risk of failure, Seifi et al. [3] recommended an IT of 5–10 Ncm, advising its use for a 1.6 mm diameter mini-implant, as higher values may lead to mini-implant fracture.

IT quantifies the frictional resistance between the screw threads and the surrounding bone, serving as a key parameter for assessing the mechanical stability of orthodontic mini-implants [4]. The highest torque recorded during insertion, expressed in Newton-centimeters (Ncm), is referred to as maximum IT, which is widely used as an indicator of initial stability in both clinical and experimental studies [5]. Research on dental implants suggests that optimizing peak IT can enhance success rates by minimizing micromotion [6]. However, excessive loading on the bone can cause structural damage and impair osseointegration during mini-implant placement [7]. Studies on orthodontic mini-implants have also reported that excessively high IT values and over-tightening can contribute to cortical bone fractures [8] and a decrease in implant retention [9]. The literature exhibits a wide range of reported fracture torques during mini-implant placement, spanning from 9.6 to 99.15 Ncm. Motoyoshi et al. [10] noted peak IT between 9.6 and 26 Ncm, while Leles et al. [11] reported a mean final IT of 55.8 ± 18.4 Ncm, with varying percentages falling within different torque ranges. Pithon et al. [12] found fracture torque values ranging from 49.6 to 99.15 N/cm^2^ across different mini-implant brands. Several factors contribute to variations in torque values during mini-implant placement and fracture, including differences in implant type, design, material, bone density, thickness, and drilling protocols. Implant surface treatment and diameter also affect mechanical properties. Understanding these factors is essential for optimizing mini-implant stability and success in orthodontics. Studies focus on mechanical strength measures such as IT [13], removal torque (RT) [14], and pullout strength [15].

Mini-implants have achieved success due to their minimally invasive nature, facilitating easy and less discomforting insertion and removal processes for patients. Moreover, they are cost-effective and offer versatile treatment options by allowing for immediate loading [16].

The clinical effectiveness of using mini-implants is well-established, and further enhancements could be realized through additional research focusing on the factors associated with failure. However, it is important to acknowledge the drawbacks associated with these devices, including root injuries [17], failure [18], fracture [19], soft tissue inflammation [20], perforation of the nasal and maxillary sinus floors [21], and intense pain on the sixth day after inserting [22], which are among the most frequent complications encountered.

The clinical success rate of mini-implants in orthodontics exceeds 80%, a figure that has improved considerably over the years, with some studies indicating survival rates that can exceed 90% in specific scenarios [23,24].

Mini-implants can experience different forces in clinical settings depending on their location and attachment to the orthodontic appliance. They are commonly placed in the buccal cortical plate in the maxilla and mandible, maxillary palate, palatal aspect of the maxillary alveolar process, and retromolar area of the mandible [25].

This study aims to compare the IT of different orthodontic mini-implants and investigate the correlation between IT, implant diameter, and length. By analyzing these relationships, this study seeks to determine how implant dimensions influence mechanical stability, providing insights for optimizing mini-implant selection in clinical practice. The null hypothesis is that IT does not differ significantly based on the type of mini-implant used.

## 2. Materials and Methods

### 2.1. Ethical Clearance

This study received approval from the Research Ethics Committee of Grigore T. Popa University of Medicine and Pharmacy Iași under approval number 178/02.05.2022. Additionally, the Committee authorized the participant information and consent forms utilized in the research.

### 2.2. Pilot Study Design

In this pilot study, we utilized 12 self-drilling orthodontic mini-implants sourced from 3 different manufacturers (Jeil Medical Corporation^®^ Seoul, Korea; Leone S.p.A, Firenze, Itlay; Lomas Dental Corporation San Diego, CA, USA). The sample size was determined based on practical factors and prior experience in the field.

The mini-implants were divided into four groups based on their diameters and lengths:

Group 1: Lengths of 6.0 mm with varying diameters (1.4, 1.6, and 2.0 mm);
Group 2: Lengths of 8.0 mm with varying diameters (1.4, 1.6, and 2.0 mm);
Group 3: Lengths of 10.0 mm with varying diameters (1.4, 1.6, and 2.0 mm);
Group 4: Lengths of 12.0 mm with varying diameters (1.4, 1.6, and 2.0 mm).

### 2.3. Artificial Bone Model and Mechanical Testing Standards

**Figure 1:**
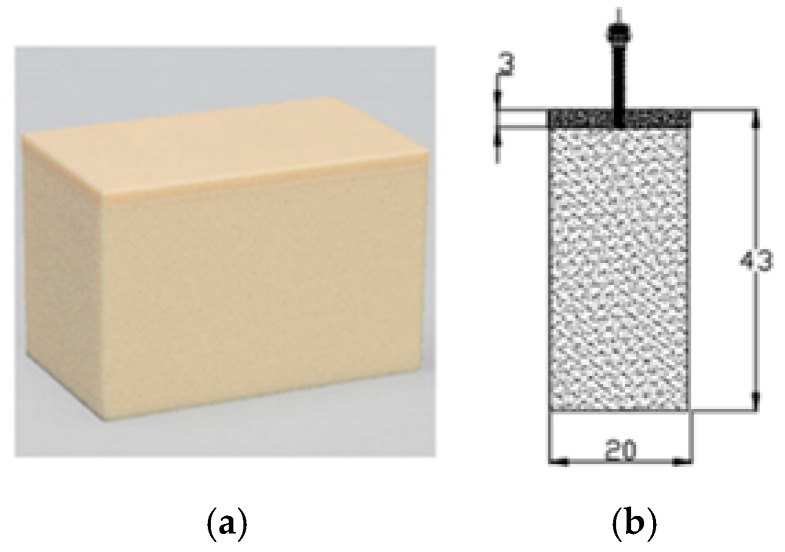
Sample with (**a**) artificial bone blocks, and (**b**) the dimensions of the blocks within the experiments the mini-implants were inserted at 90°.

The mini-implants were inserted at 90°, without predrilling, into artificial bone blocks (Sawbones^®^, Vashon Island, WA, USA) (Figure 1a,b) and were complied with the specifications of the American Society for Testing and Materials. Although this material does not replicate human bone structures, it exhibits mechanical properties within the range of the human cancellous bone density, as described in the ASTM F-1839-08 standard [26].

A greater thickness of artificial bone may contribute to better mechanical stability in the model, providing a more robust environment for evaluating the performance of the mini-implants without being influenced by the potential fragility of the thinner layers of artificial bone. Pan et al. reported that the average cortical bone thickness ranges from 1.09 to 2.12 mm in the maxilla and from 1.59 to 3.03 mm in the mandible, with the mandible generally having a thicker cortical bone [27].

The mechanical properties of the artificial bone are expressed in units such as g/cc (density) and MPa (strength and modulus of compression, tension, and shear). These mechanical characteristics of the artificial bone can influence the behavior of a mini-implant when inserted into this type of material. The data on artificial bone (Table 1) present the density, compression, tension, and shear values at different fractional volumes. The specific properties of the bone, as presented in Table 1, can influence the mini-implant’s ability to achieve initial stability, withstand loads during orthodontic treatment, and undergo effective osseointegration.

Based on these findings, we selected an artificial bone model with a 3 mm thick 40 PCF solid foam laminated on one side of the block (Figure 1b) to closely simulate the cortical bone thickness in the mandible.

**Figure 2:**
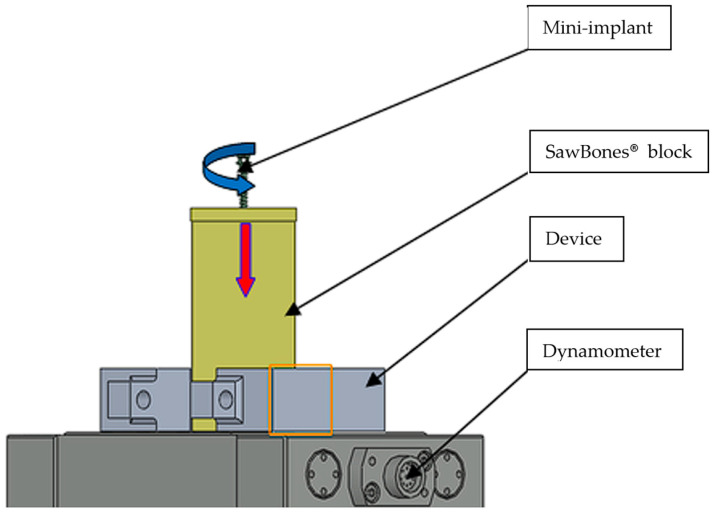
Drawing of the device for bone blocks that are threaded and clamped onto the dynamometer with a jaw device.

The IT for each mini-implant was experimentally assessed according to the ASTM F543-A2 method [29]. The aim was to ensure the conditions for obtaining precise and comparable data to formulate clear and fair conclusions regarding the studied phenomenon or process (Figure 2). Additionally, precision in measurement was pursued, ensuring that the results of the investigations were relevant and representative of practical applications.

### 2.4. Data Acquisition and Biomechanical Analysis of Insertion Force (IF) and Torque

**Figure 3:**
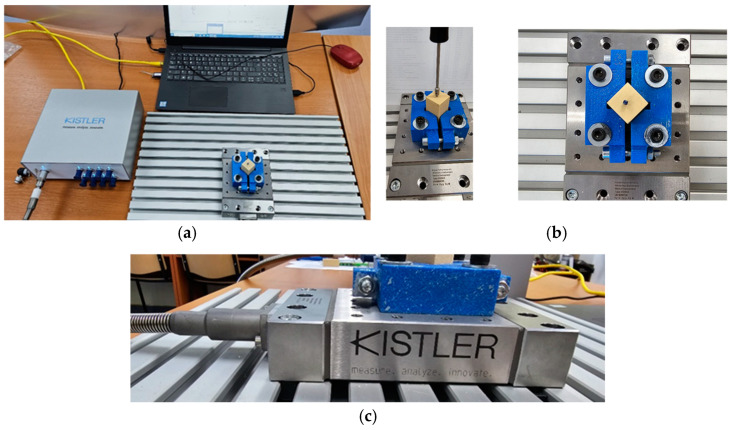
The data acquisition system: (**a**) The data acquisition system includes the Kistler 9129AA piezoelectric dynamometer connected to a Kistler Labamp 5167A amplifier and a computer running DynoWare 2825A software for real-time force and torque analysis. (**b**) Close-up of the experimental setup showing a mini-implant inserted into a 20 mm × 20 mm artificial foam block secured in a holding mechanism. (**c**) Side view of the Kistler 9129AA dynamometer illustrating its precise alignment and connection for accurate measurement during testing.

The IF and IT were measured using a data acquisition system composed of a piezoelectric multi-component dynamometer, specifically the Kistler 9129AA (Figure 3a,c) (Kistler AG, Winterthur, Switzerland), connected to a multichannel amplifier, the Kistler Labamp Type 5167A (Kistler AG, Winterthur, Switzerland), for measurements via computer, and a computer running the DynoWare 2825A software, version 5697A1 (Figure 3b) (Kistler AG, Winterthur, Switzerland).

The signals from the piezoelectric dynamometer were simultaneously directed to the data acquisition system, which allows for the direct acquisition of values for the IF and IT. A solid foam block for biomechanical testing and product demonstration was used to measure the force and torque of insertion for various orthodontic implants. The foam block material has a basic size of 170 mm × 120 mm × 43 mm, from which test blocks with sides of 20 × 20 were detached and used in the experiments. The depiction of the experimental data obtained for the Lomas Mondefit-2.00 × 12 mm mini-implant exhibits the following features. Change over time or index: The number of readings taken during the determination is displayed. The red curve represents the evolution of the measured force (p) on the z/vertical axis by the transducer, whereas the blue curve represents the evolution of the measured torque.

**Figure 4:**
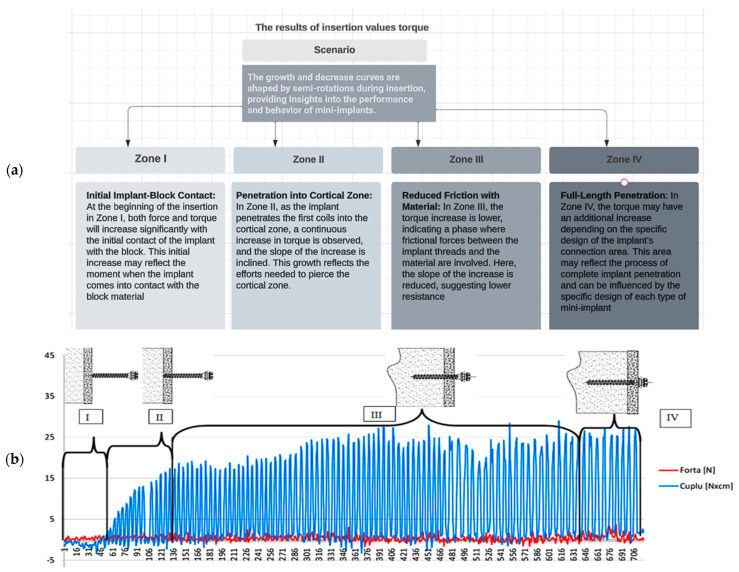
(**a**) The interpretation of the results for different sizes of mini-implants, considering the curves of force and torque evolution concerning the number of readings. (**b**) View of the experimental results for the Lomas Mondefit 2 × 8.0 mm mini-implant.

In the evolution of the two curves, the four zones were delineated as follows (Figure 4):

Zone I—Where the contact between the implant and the block occurs;
Zone II—After penetrating the first coils of the implant into the cortical zone, an increase in torque can be observed until piercing this zone, and the slope of the increase inclines;
Zone III—An area where the increase in driving torque due to friction between the implant’s flanks and the material has a very low slope of increase;
Zone IV—The zone where the implant has penetrated the entire length and, depending on the shape of the implant’s connection area (between the cylindrical zone and the head of the implant), there may be an additional torque increase. The shape of the curves with a growth–decrease pattern is caused by each semi-rotation of the implant/screwdriver used.

The final insertion depth was measured after each test, and the relationships between the torque and rotation obtained from the torque sensor were converted into relationships between torque and insertion depth. Insertion tests were repeated twice for each mini-implant to account for experimental variability. A new mini-implant was used for each repetition to ensure uniform testing conditions and minimize any potential alterations in the implant structure or insertion material from previous tests.

### 2.5. Data Analysis

The collected data underwent analysis using SPSS Software (Version 20.0) (SPSS^®^ Inc., Chicago, IL, USA) to generate descriptive statistics and conduct data analysis. Descriptive statistics encompassed frequency, percentages, means, and standard deviations. A *p*-value of <0.05 was considered significant. One-way analysis of variance with a post hoc Tukey’s honest significant difference test was used for intergroup comparisons and ANOVA results for checking significant differences in insertion based on the type of mini-implant. The Shapiro–Wilk test was conducted for each group of data. The results indicate that the data were normally distributed (*p* > 0.05), justifying the use of an ANOVA.

## 3. Results

### 3.1. Comparative Analysis of IF and Torque Across Mini-Implant Types and Sizes

Table 2 summarizes the IT results of average IF and average IT for different mini-implant sizes. In the insertion on the 90° test, the IT of Group 2 (35 N/cm) was significantly higher than that of Group 1 (14 N/cm). The analysis sought to identify any significant differences among types of mini-implants and investigate potential correlations with the mini-implants’ diameters and lengths. Significant differences were observed within Group 2, particularly in the case of the 2 × 8 mini-implant manufactured by Lomas Mondefit; this particular mini-implant showing elevated average IT = 35 N when compared to other mini-implants in the same group. This implies a potentially higher IT, signifying variations in mechanical performance among different types within this specific group. Additionally, in Group 3, the 2 × 10 mini-implant from Lomas Mondefit exhibits higher AIF = 28 compared to others in the same group, indicating potential differences in IT.

After conducting an in vitro analysis on the maximum IT values for various types of orthodontic mini-implants from different manufacturing brands, the obtained results are as follows: a significant variation in maximum IT values was observed among the three types of mini-implants (Jeil 2 × 6, AIT = 25; Lomas Mondefit 2 × 8, AIT = 35; Lomas Mondefit 2 × 12, AIT = 23), as well as correlation between torque and diameter: as the diameter increases, a concomitant augmentation in torque is observed (Jeil 1.4 × 6, AIT = 14 and Lomas Mondefit 2 × 12, AIT = 23).

### 3.2. Statistical Analysis: Analysis of Insertion Differences Among Types of Mini-Implants

A descriptive analysis of the variables for each type of mini-implant: The calculation of Spearman’s rank correlation coefficient and the p-test aims to identify any significant differences between the types of mini-implants and investigate the potential correlations with the diameter and length of the mini-implant (Table 3). For all groups, it is observed that the mean values are close to the median values, indicating the absence of outliers in the three groups for both types of insertion. Increased homogeneity among the groups is also evident when analyzing the standard deviation values, which are less than half of the mean value, suggesting that there are no significant differences in insertion among the units within each group.

### 3.3. Analysis of Insertion Differences Among Types of Mini-Implants

a. An evaluation of maximum IT was performed using a measurement and comparison of the maximum IT values for the three types of orthodontic mini-implants in order to identify and quantify the differences in the mechanical performances of these mini-implants concerning IT.

The null hypothesis is that IT does not differ significantly based on the type of mini-implant used. The probability value (Sig.) is <0.05 only for Average IT (Sig. = 0.035), (Table 4) indicating that Average IT differs based on the type of mini-implant used, meaning that there are at least two types of mini-implants for which the average IT is different.

Using Tukey’s post hoc test (Table 4), we verified these insertion differences for all pairs of mini-implants to observe which types of implants show significant differences in insertion. It was observed that the probability value is less than 0.05 only for the comparison between LEONE and LOMAS VEGAS (*p* = 0.029) (Table 5), signaling that there is a significant difference in insertion only between these two implant types.

b. Correlation of maximum IT with mini-implant diameter.

To investigate the existence of a correlation between the values of maximum IT and the diameter of the mini-implant to provide information on how the diameter of the mini-implant may influence the level of IT, contributing to a deeper understanding of their performance, we used the Spearman correlation coefficient. The results indicate a direct and moderately strong relationship (0.609), meaning that as the diameter increases, so does the insertion (Table 6).

c. An analysis of the influence of the mini-implant’s design on IT was performed by examining how the design of the mini-implant can affect the values of IT and its stability. We try to identify design factors that can influence the mechanical performance of mini-implants and provide useful information for their improvement. By achieving these specific objectives, the analysis aims to make a significant contribution to the existing knowledge about the mechanical characteristics of orthodontic mini-implants.

The Spearman correlation coefficient was calculated to assess the relationship between insertion and the length of the mini-implant, and the results show, for *p* less than 0.05, a direct and very strong relationship between insertion and length of the mini-implants (0.890), indicating that if the length of mini implants increases, so does the IF (Table 7).

## 4. Discussion

The analysis of the results from this study provides comprehensive insights into the mechanical performance of orthodontic mini-implants, highlighting several key findings. Firstly, significant variations in average IT were observed among different types of mini-implants, particularly evident in Lomas Mondefit implants within groups 2 and 3. Specifically, the 2 × 8 mini-implant from Lomas Mondefit exhibited notably higher average IT compared to other mini-implants in its respective groups, indicating variability in mechanical performance among specific categories. Furthermore, this study conducted an in vitro analysis on maximum IT values across different mini-implant types and manufacturers. The results indicate considerable diversity in maximum IT values among the three types studied (Jeil 2 × 6.0 mm, AIT = 25; Lomas Mondefit 2 × 8.0 mm, AIT = 35; Lomas Mondefit 2 × 12.0 mm, AIT = 23), highlighting the significant influence of mini-implant type on mechanical stability during insertion procedures. Correlation analyses further revealed a direct and moderately strong relationship between IT and mini-implant diameter (Spearman’s rho = 0.609). This finding suggests that larger diameter mini-implants tend to require higher IT, emphasizing the role of diameter in enhancing mechanical stability during placement. The thickness of the cortical bone plays a significant role in determining implant IT, with thicker cortical bones leading to higher torque values [30].

The survival rate of mini-implants is significantly influenced by the IT, with higher torque values potentially leading to stress, necrosis, and local ischemia [31]. The diameter of the mini-implants significantly influenced the IT values. Studies have shown that larger diameter mini-implants, such as 2 mm, exhibit significantly higher IT than smaller diameters, such as 1.6 mm [32]. Additionally, the diameter and thread design of mini-implants has been identified to have a distinct impact on primary stability, indicating that variations in diameter can influence the IT [33]. Research has also demonstrated significant differences in insertion and extraction torques among different implant diameters, with an increasing trend observed for larger diameters [34]. Furthermore, it has been concluded that, with an increase in the diameter of a mini-implant, there is a greater contact surface between the mini-implant and bone, resulting in improved implant stability [35]. Collectively, these findings indicate that the diameter of mini-implants plays a crucial role in influencing IT values, with larger diameters generally associated with higher IT and improved stability.

Moreover, the correlation analysis between IT and mini-implant length showed a strong positive relationship (Spearman’s rho = 0.890). This indicates that longer mini-implants generally necessitate higher IT for effective placement, highlighting the critical role of implant length in achieving adequate mechanical stability. The length of mini-implants had a significant influence on IT values. Studies have demonstrated a positive correlation between mini-implant length and IT, with longer implants typically exhibiting a higher IT [36]. Additionally, the length of the mini-implant has been identified as a significant factor that distinctly affects measured IT values, with greater insertion depths resulting in higher IT [37]. Moreover, one study highlighted that increasing the length of mini-implants leads to improved stability, indicating a positive correlation between length and stability [38]. Furthermore, the results of a comparative study indicate a direct relationship between maximum torque and mini-implant length, emphasizing the significant impact of length on IT values [12]. The length of mini-implants significantly influences IT values, with longer implants being generally associated with higher IT and improved stability. The assessment of IT in mini-implants involves various techniques. The ANOVA test for Insertion Torque (IT) reveals a *p*-value of 0.035, which is statistically significant (*p* < 0.05).

This suggests that the type of mini-implant has a significant effect on insertion torque, meaning at least one group differs significantly from the others.

These include using a torque measurement device to evaluate the mechanical response of mini-implants during insertion and extraction, allowing for the measurement of consecutive torques at specific time intervals [39]. Additionally, studies have employed surgical motors to record IT at precise intervals during mini-implant placement, providing valuable data on the torque experienced during insertion [40]. Moreover, the IT was assessed using a torque-indicating ratchet wrench, allowing for the measurement of torque values required for drilling mini-implants into simulated bone blocks [19]. Furthermore, the IT was evaluated by comparing the torque values for different groups of mini-implants through statistical analyses such as ANOVA to determine significant differences [41]. Atieh et al. [42] conducted a comprehensive review and meta-analysis to assess the effects of IT values on dental implant outcomes. Their findings suggest that utilizing either high or regular IT during implant placement resulted in comparable short-term failure rates, changes in marginal bone levels, and complication rates. ANOVA results indicate significant differences in average IT among different mini-implant types, particularly between Leone and Lomas Mondefit. Spearman correlation coefficients demonstrated a significant correlation between IT and both the diameter and length of the mini-implants.

Based on the initial assumption that IT does not vary significantly based on the type of mini-implant used, this study’s results and discussions yield several conclusive insights: This research uncovered notable differences in average IT among various types of orthodontic mini-implants, particularly with Lomas Mondefit implants from groups 2 and 3. Specifically, the 2 × 8 mini-implant exhibited markedly higher IT compared to others within its category, indicating significant mechanical performance discrepancies within specific mini-implant groups. Furthermore, the in vitro analysis revealed substantial diversity in maximum IT values across different mini-implant types and manufacturers. Notably, this study underscores the substantial influence of mini-implant type on mechanical stability during insertion procedures. Correlation analyses further underscored the impact of diameter and length on IT. They revealed a direct and moderately strong relationship between IT and mini-implant diameter, suggesting that larger diameters typically require higher IT for effective placement. Similarly, a robust positive correlation was observed between IT and mini-implant length, highlighting the critical role of implant length in achieving sufficient mechanical stability.

Since the *p*-values for both variables in Levene’s test are greater than 0.05, we conclude that there are no significant differences in the variances between the groups for IF and IT. Thus, we can assume that the variances are equal (homoscedasticity), allowing for the use of parametric tests for comparative analysis.

The factors influencing the maximum IT values of mini-implants are complex and encompass various aspects. Additionally, the design of mini-implants, such as the presence of cylindrical implants with two threads, significantly affects both insertion and extraction torques, thus influencing stability [43]. Moreover, the maximum IT can be influenced by the impact of the implant flange on the serrated cortical bone, the ‘bottoming out’ of the implant, and implant engagement [44]. The fracture resistance of mini-implants varies depending on the manufacturer and type, necessitating the knowledge of these characteristics before clinical use [45]. Furthermore, the presence of a pilot hole has minimal effect on the microdamage characteristics and maximum IT [46]. Geometric design features, such as the thread form factor, play a key role in maintaining primary stability [47]. The characteristics of mini-implants that influence the maximum IT values cover a range of factors, including design, surface characteristics, and the presence of a pilot hole. Collectively, these factors contribute to the primary stability and survival rates of mini-implants, emphasizing the importance of considering these features in clinical practice.

### 4.1. Limitations of the Study

This in vitro analysis evaluates the maximum IT of orthodontic mini-implants using an artificial bone model (Sawbones^®^). Although this controlled setting enabled accurate measurements, the results may not entirely represent clinical situations, where factors like varying bone density and tissue composition can influence IT.

- Limited sample size: This study examines a total of 12 mini-implants from three different manufacturers, categorized into four groups based on diameter and length. While this provided valuable insights into the mechanical properties of these specific mini-implants, a larger sample size and inclusion of additional brands could offer a more comprehensive understanding of IT variability.
- Lack of pre-drilling: Mini-implants were inserted at a 90° angle into the artificial bone without pre-drilling. While this simulated clinical conditions where pre-drilling may not always be feasible, it could have influenced the IT values observed. Pre-drilling is a common clinical practice that can impact the stability and torque values of mini-implants.
- Single torque measurement: This study focused solely on maximum IT values and did not assess removal torques or mechanical stability over time. Evaluating both insertion and removal torques, as well as assessing mechanical stability under loading conditions, would provide a more comprehensive understanding of mini-implant performance.
- Manufacturer-specific differences: Variability in mini-implant design and manufacturing processes among different brands may have influenced the observed torque values. While this study compares mini-implants from three manufacturers, additional factors such as surface treatment and material composition could also contribute to torque variability, but were not addressed in this analysis.

### 4.2. Future Directions

- Randomized controlled trials (RCTs): Conducting RCTs will help validate these findings in real clinical scenarios, ensuring a higher level of evidence.
- Comparative studies: Future research should compare mini-implants with alternative anchorage devices to determine the most effective approach in different orthodontic cases.
- Long-term clinical validation: Future studies should investigate the long-term stability and success rates of different mini-implant designs in in vivo conditions.

## 5. Conclusions

This study on orthodontic mini-implants provides significant findings regarding their mechanical performance, highlighting the variability in IT values among different types and manufacturers. The results underscore the critical roles of diameter and length in influencing IT, with larger diameters and greater lengths generally requiring higher torques for successful placement. While this study offers valuable data on IT characteristics, it is important to consider limitations such as the use of an artificial bone model and sample size constraints when interpreting the results. Addressing these limitations in future research could further enhance our understanding of mini-implant stability and performance in clinical settings.

## Figures and Tables

**Table 1:** Mechanical properties of artificial bone [28].

Density			Compression		Tension		Shear	
PCF	g/cc	Fractional Volume	Strength MPa	Modulus MPa	Strength MPa	Modulus MPa	Strength MPa	Modulus MPa
20	0.32	0.27	8.4	210	5.6	284	4.3	49
40	0.64	0.54	31	759	19	1000	11	130

**Table 2:** The results of the average IF and torque for different implant sizes.

Group	Diameter (mm)	Type	Manufacturer	IF (N)	IT (N/cm)
1: Length 6 mm	1.4	Self-drilling	Jeil	1.2–1.5	12–14
	1.6	Self-drilling	Jeil	1.8–2	16–17
	2.0	Self-drilling	Jeil	2.3–2.4	25
2: Length 8 mm	1.4	Self-drilling	Leone	2.26–2.8	18–19
	1.6	Self-drilling	Jeil	2.7	28–30
	2.0	Self-drilling	Lomas	3.6–3.8	34–35
3: Length 10 mm	1.4	Self-drilling	Jeil	3.5	20–22
	1.6	Self-drilling	Leone	3.5	18
	2.0	Self-drilling	Lomas	3.0	27–28
4: Length 12 mm	1.4	Self-drilling	Lomas	4.3–5.0	20–23
	1.6	Self-drilling	Leone	4.7	13–15
	2.0	Self-drilling	Jeil	4.5	20–23

**Table 3:** Descriptive indicators for insertion variables for each type of mini-implant.

Manufacturer	Indicator	Mean	Median	Standard Deviation
Jeil	Average IF	2.850	2.550	1.2502
	Average IT	21.83	22.50	5.707
Leone	Average IF	3.633	3.500	1.0066
	Average IT	17.00	18.00	1.732
Lomas	Average IF	3.400	3.400	0.5657
	Average IT	31.50	31.50	4.950

**Table 4:** ANOVA results for checking significant differences in insertion based on the type of mini-implant.

		Sum of Squares	df	Mean Square	F	Sig.
IF	Between Groups	1.364	2	0.682	0.537	0.604
	Within Groups	10.162	8	1.270		
	Total	11.525	10			
IT	Between Groups	254.848	2	127.424	5.273	0.035
	Within Groups	193.333	8	24.167		
	Total	448.182	10			

**Table 5:** Tukey test results for multiple comparisons.

Variabila	JEL	LEONE	LOMAS
Jeil		0.390	0.097
Leone	0.390		0.029 *
Lomas	0.097	0.029 *	-

* Significant difference for *p* < 0.05.

**Table 6:** Results of the correlation between IT and diameter (Spearman’s Rho).

Variable	IF—Correlation Coefficient	IF—Sig. (2-Tailed)	IT—Correlation Coefficient	IT—Sig. (2-Tailed)	Diameter—Correlation Coefficient	Diameter—Sig. (2-Tailed)
IF	1.000	-	0.265	0.430	0.586	0.058
IT	0.265	0.430	1.000	-	0.609 *	0.047 *
Diameter	0.586	0.058	0.609 *	0.047 *	1.000	-

* Significant difference for *p* < 0.05.

**Table 7:** Results of the correlation between insertion and length (Spearman’s Rho).

Variable	IF—Correlation Coefficient	IF—Sig. (2-Tailed)	IT—Correlation Coefficient	IT—Sig. (2-Tailed)	Length—Correlation Coefficient	Length—Sig. (2-Tailed)
IF	1.000	-	0.265	0.430	0.890 *	0.000 *
IT	0.265	0.430	1.000	-	0.056	0.869
Length	0.890 *	0.000 *	0.056	0.869	1.000	-

The results of the Levene’s test for evaluating the variances of IF and IT between groups are as follows: IF—Levene statistic = 0.186, *p*-value = 0.902; IT—Levene statistic = 0.181, *p*-value = 0.906. * Significant difference for *p* < 0.05.

## Data Availability

Data are contained within the article.
